# A proposed mechanism for nitrogen acquisition by grass seedlings through oxidation of symbiotic bacteria

**DOI:** 10.1007/s13199-012-0189-8

**Published:** 2012-10-05

**Authors:** James F. White, Holly Crawford, Mónica S. Torres, Robert Mattera, Ivelisse Irizarry, Marshall Bergen

**Affiliations:** 1 Department of Plant Biology and Pathology, Rutgers University, New Brunswick, NJ USA; 2 Department of Engineering Technologies, Safety, and Construction, Central Washington University, Ellensburg, WA USA

**Keywords:** Grasses, Nitrogen fixation, Nitrogen scavenging, Reactive oxygen species, Symbiosis

## Abstract

In this paper we propose and provide evidence for a mechanism, oxidative nitrogen scavenging (ONS), whereby seedlings of some grass species may extract nitrogen from symbiotic diazotrophic bacteria through oxidation by plant-secreted reactive oxygen species (ROS). Experiments on this proposed mechanism employ tall fescue (*Festuca arundinaceae*) seedlings to elucidate features of the oxidative mechanism. We employed 15N_2_ gas assimilation experiments to demonstrate nitrogen fixation, direct microscopic visualization of bacteria on seedling surfaces to visualize the bacterial oxidation process, reactive oxygen probes to test for the presence of H_2_O_2_ and cultural experiments to assess conditions under which H_2_O_2_ is secreted by seedlings. We also made surveys of the seedlings of several grass species to assess the distribution of the phenomenon of microbial oxidation in the Poaceae. Key elements of the proposed mechanism for nitrogen acquisition in seedlings include: 1) diazotrophic bacteria are vectored on or within seeds; 2) at seed germination bacteria colonize seedling roots and shoots; 3) seedling tissues secrete ROS onto bacteria; 4) bacterial cell walls, membranes, nucleic acids, proteins and other biological molecules are oxidized; 5) nitrates and/or smaller fragments of organic nitrogen-containing molecules resulting from oxidation may be absorbed by seedling tissues and larger peptide fragments may be further processed by secreted or cell wall plant proteases until they are small enough for transport into cells. Hydrogen peroxide secretion from seedling roots and bacterial oxidation was observed in several species in subfamily Pooideae where seeds possessed adherent paleas and lemmas, but was not seen in grasses that lacked this feature or long-cultivated crop species.

## Introduction

Non-pathogenic microbes are abundant on plants as epiphytes and endophytes (Klein et al. 1990; Stone et al. 2000; Tsavkelova et al. 2004). The benefits to plants that host these microbes are generally subtle, but they are gradually being discerned and clarified (Redman et al. 2002; Rodriguez et al. 2009; Puente et al. 2009; Torres et al. 2012). Often benefits are found to be defensive with microbes protecting plants from biotic stresses (White et al. 2001; Clay et al. 2005; Clarke et al. 2006; Selosse and Schardl 2007; Álvarez-Loayza et al. 2011; Bacon and Hinton 2011) or abiotic stresses (Redman et al. 2002; Malinowski et al. 2005; Waller et al. 2005; Kuldau and Bacon 2008; White and Torres 2010; Torres et al. 2012; Hamilton et al. 2012). Some bacterial epiphytes and endophytes are capable of nitrogen fixation (Reinhold-Hurek et al. 1993; Döbereiner 1992; Döbereiner et al. 1994; James et al. 1994; Hurek et al. 1994; Kloepper 1994; Puente and Bashan 1994; Feng et al. 2006; Magnani et al. 2010). Many of these microbes stimulate development of the plant host (e.g. Plant-Growth Promoting Rhizobacteria (PGPR)) (Kloepper 1993, 1994).

It has been proposed and often assumed that nitrogen fixed from the atmosphere by endophytic or epiphytic microbes may be utilized by host plants for growth (Döbereiner et al. 1994; Dakora et al. 2008). However, these microbes are frequently capable of producing plant growth regulators such as auxins and cytokinins (e.g., Glick 1995; Feng et al. 2006). Because of the production of plant growth regulators by many symbiotic microbes of plants, growth stimulation effects due to diazotrophic microbes may be attributed to effects of growth regulators rather than to nitrogen fixation by microbes (e.g., Bashan et al. 1989; Mantelin and Touraine 2004; Feng et al. 2006; Ortíz-Castro et al. 2008). The absence of any clear mechanism by which nitrogen fixed by bacteria may be transferred to the plant host further adds to the uncertainty as to whether plants obtain nitrogen from endophytic or epiphytic diazotrophic bacteria (Lethbridge and Davidson 1983; James and Olivares 1998; James 2000; Steenhoudt and Vanderleyden 2000). Without a nitrogen transfer mechanism, nitrogen assimilated into organic molecules by the bacterial populations on plants may simply remain in the microbial nitrogen pool rather than move to plants to support plant growth (Bremer et al. 1995; James and Olivares 1998; Zogg et al. 2000; James 2000). This is due in part to the nitrogen scavenging capacity of bacteria and the tenacity by which bacterial populations within or on the surfaces of plants hold onto fixed nitrogen (Hill and Postgate 1969; Hurek et al. 1988; Steenhoudt and Vanderleyden 2000). In this paper we report work we have conducted on grass seedlings with associated seed-transmitted bacteria and provide evidence for a mechanism for the transfer of fixed nitrogen from bacteria to seedlings via oxidation of the microbes and/or their protein products. We term this mechanism, oxidative nitrogen scavenging (ONS).

## Materials and methods

### Source of tall fescue seeds

For experiments described in 2.2–2.6 below, we used one-year old seeds of tall fescue grass (*Festuca arundinaceae*) that were originally collected in Morocco and subsequently maintained at the Rutgers University Turfgrass Research Station in Adelphia, New Jersey. We replicated experiments using various commercially available tall fescue seeds to confirm the presence of diazotrophic bacteria on seeds and H_2_O_2_ secretion from seedlings.

### Isolation and identification of bacteria from tall fescue seeds

To isolate and identify bacteria, we placed 4 non-disinfected seeds (caryopses) onto each of five petri plates (15 cm. diam.) containing potato dextrose agar (PDA; Difco, Becton, Dickenson and Company, Sparks, MD). Using 980 base pairs of the 16S rDNA region (Baker et al. 2003) we identified four of five morphologically unique isolates to *Pantoea agglomerans*; and a fifth isolate to *Pseudomonas* sp. We submitted representative sequences to NCBI (GenBank JX089400 and JX089401).

### Effects of light and bacteria on H_2_O_2_ secretion of tall fescue seedlings

To assess the effect of light and microbial presence on seedling H_2_O_2_ secretion*,* we conducted peroxide secretion experiments using the following treatments: 1.) 12 h alternating light/dark with surface disinfected seeds; 2.) 12 h alternating light/dark with non-disinfected seeds; 3.) dark (24 h) with surface disinfected seeds; 4.) dark (24 h) with non-disinfected seeds. In each of these treatments we used 5 plates (approximately 20 seeds/plate) containing 1 % agarose media. For the disinfected seed treatments, seeds were disinfected for 30 min using 50 % Clorox® followed by a 30 min rinse in sterile distilled water. We incubated all plates at ambient temperature for 7 days to facilitate seed germination. On the eighth day after plating, we flooded plates with 5 ml solution of 100 mM potassium phosphate buffer, pH 6.9, 2.5 mM diaminobenzidine tetrachloride and 5 purpurogallin units/ml of horseradish peroxidase (Type VI, Sigma Chemical Company, St. Louis, MO) (Munkres 1990; Pick and Keisari 1980). Plates were incubated at 30 °C in dark for 1 h before the solution was discarded. After 10 h, we rinsed the plates in sterile, distilled water, and examined them for red diffusible zones due to H_2_O_2_ around the seedlings. We then rated the intensity of the H_2_O_2_ zones as follows: 0 = no zones; + = low intensity or lighter zones; ++ = moderate intensity zones; +++ = high intensity or larger zones).

### Effects of nutrient environment on H_2_O_2_ secretion of tall fescue seedlings

We also sought to determine the effect(s) of the medium nutrient environment on seedling H_2_O_2_ secretion. We amended 1 % (w/v) agarose (Type 1 Low EEO; Sigma-Aldrich, St. Louis, MO) media with 0.01 % (w/v) and 0.1 % (w/v), respectively, of L-alanine (Sigma, St. Louis, MO), glycine (Sigma, St. Louis, MO), yeast extract (Bacto™; Difco, Becton, Dickenson and Company, Sparks, MD), ammonium nitrate (Sigma, St. Louis, MO), sodium nitrate (Sigma, St. Louis, MO), and ammonium phosphate (Dibasic; Fisher Scientific Company, Fair Lawn, NJ). Media amendments were added after autoclaving to avoid thermal decomposition of amendments. In this experiment we replicated each treatment using 5 plates (20 seeds that had not been surface disinfected on each plate). In a separate experiment we prepared two sets of 5 plates: 1) 1 % (w/v) agarose media; and 2) 1 % agarose amended with 0.1 % (w/v) cellulase enzyme (from *Aspergillus niger*; Sigma, St. Louis, MO). To inactivate the cellulase enzyme we boiled the medium for 30 min prior to cooling and pouring into plates. In this experiment we used seeds that had been surface disinfected (see Section 2.3) to reduce bacterial populations on seedlings. After 7 days of incubation at laboratory ambient temperature with a 12 h alternating light/dark cycle, we stained the plates with DAB/horseradish peroxidase and evaluated them as described in Section 2.3 above. These results are summarized in Table 1.

**Table 1:** Summary of substrate effects on seedling secretion of hydrogen peroxide into agarose media

Agarose medium amendment	Seed treatment prior to germination	Percentage of seedlings showing H_2_O_2_ secretion zones^a^	Average intensity of H_2_O_2_ zones^b^
None^c^	Disinfection	23.08	+
None	None	100	+++
Yeast Extract (0.01 %)	None	100	+++
Yeast Extract (0.1 %)	None	54	++
Alanine (0.01 %)	None	96.3	+++
Alanine (0.1 %)	None	21.05	+
Glycine (0.01 %)	None	88	+++
Glycine (0.1 %)	None	40	+
Inactivated Cellulase (0.1 %)	Disinfection	100	+++
Ammonium Nitrate (0.01 %)	None	86.2	+++
Ammonium Nitrate (0.1 %)	None	78.13	+++
Potassium Nitrate (0.01 %)	None	76.67	+++
Potassium Nitrate (0.1 %)	None	86.67	+++

^a^Average of 100 seedlings assessed

^b^+ = low intensity or lighter zones, +++ is higher intensity or darker zones, and ++ are intermediate intensity zones

^c^Control from cellulase enzyme amendment experiment

### Visualization of bacterial oxidation on tall fescue seedlings

To visualize bacterial oxidation on seedlings, we stained plates bearing seedlings with DAB/horseradish peroxidase for a 10 h period. We then excised seedling roots and shoots, placed them on a slide containing 1 % aniline blue/lactic acid stain, and examined the slide using bright field microscopy (Bacon and White 1994). On a second set of slides, we stained seedling roots (to visualize nucleic acids during oxidation) with SYTO9® (Life Technologies, Carlsbad, CA) and observed them using fluorescence microscopy on a Zeiss Axioskop (using the FITC filter system (range 430–520 nm for Fig. 2a and b; and blue violet excitation (range 395–440 nm) for Fig. 2c and d).

### Nitrogen assimilation of tall fescue seedlings

To assess nitrogen assimilation by seedlings, we placed surface-disinfected seeds or non-disinfected seeds (approximately 20/plate) onto 5 petri plates for each treatment containing 1 % (w/v) agarose media, and then placed the plates into a 1-liter nitrogen assimilation chamber (constructed using a sealable glass jar with gas input ports) into which we added 15N_2_ gas and incubated at laboratory ambient temperature. We repeated this experiment three times adding 20 mls of ^15^N_2_ gas in run 1, 125 mls of ^15^N_2_ gas in run 2, and 250 mls ^15^N_2_ gas in run 3. After a 3-week incubation period under 12 h alternating light/dark cycle we removed the plates from the chamber, washed the seedlings with sterile, distilled water and excised shoots from the seedling roots. Experimental controls included a shoot sample and three separate samples of seeds and agarose media that had not been exposed to 15N_2_ gas. For mass-spectroscopic ^14^N/^15^N ratio analysis, we oven dried all samples for 24 h at 80 °C. After drying, we sent 0.9-1.0 g of dried material to the Stable Isotope/Soil Biology Laboratory at the Odum School of Ecology at the University of Georgia for analysis (Radajweski et al. 2000). Results of the isotopic nitrogen analysis are reported in Table 2.

**Table 2:** 15N isotopic analysis results for 15N enriched and non-enriched plants after 3 weeks growth

Treatment	Sample wt (mg)	Total % nitrogen	δ^15^N vs (‰)	Atoms % ^15^N
Agarose media (no15N controls)	1.268	0	N/A^a^	N/A^a^
	1.750	0		
	1.231	0		
Seeds (no 15N controls)	1.303	2.10	0.70	0.366729
	1.082	2.00	0.64	0.366705
	1.325	2.11	0.79	0.366761
Shoots (no 15N Control)	1.102	3.82	2.02	0.367209
Run 1 sterilized (20 mls 15N gas)	1.412	3.78	17.05	0.372697
Run 1 non-sterilized (20 mls 15N gas)	1.733	3.50	23.49	0.375049
Run 2 sterilized (125 mls 15N gas)	1.091	2.95	194.18	0.437322
Run 2 non-sterilized (125 mls 15N gas)	1.049	2.91	214.09	0.444582
Run 3 sterilized (250 mls 15N gas)	1.962	4.21	152.92	0.422274
Run 3 non-sterilized (250 mls 15N gas)	1.250	3.2	385.67	0.507094

^a^Below detection limit

### Survey of grass species for seedling root secretion of H_2_O_2_ and bacterial oxidation

To evaluate how widespread the phenomenon of microbial oxidation on seedling roots is we screened seedlings of several species in agarose media (Table 3). Species screened included species in genera *Bromus*, *Festuca, Lolium, Phalaris, Poa, Secale, Sorghum, Triticum* and *Zea*. In these experiments 3–6 non-disinfected seeds of each species were placed onto each of 3 plates of agarose media. After 7–14 days of incubation at laboratory ambient temperature with a 12 h alternating light/dark cycle, we stained the plates by flooding with DAB/horseradish peroxidase. After 10 h we rinsed the plates with water, evaluated presence of H_2_O_2_ zones, and rated intensity of H_2_O_2_ zones as described in Section 2.3, above. We also noted any enhanced H_2_O_2_ staining in root or shoot tissues. We then excised seedling roots and placed them on a slide containing 1 % aniline blue/lactic acid stain. We examined root and shoot surfaces using bright field microscopy for any evidence of bacterial oxidation. Changes in bacterial shape, loss of integrity of bacterial cell walls, and loss of capacity to stain using the protein stain aniline blue was taken as evidence of bacterial oxidation. These results are summarized in Table 3.

**Table 3:** Survey of grass species for hydrogen peroxide secretion from seedlings and bacterial oxidation

Plant species/‘Common Name’	Plant family/subfamily	Collection origin/location	Typical habitat	H_2_O_2_ intensity on/or around seedling roots	Evidence of bacterial oxidation on roots
*Bromus tectorum/*‘cheatgrass’	Poaceae/Pooideae	South River, New Jersey	Meadow	+++^1^	Yes
*Festuca arundinaceae/*‘Tall fescue grass’	Poaceae/Pooideae	Adelphia, New Jersey, USA	Meadow	+++	Yes
*Festuca ovina/*‘sheep’s fescue’	Poaceae/Pooideae	Helsinki, Finland	Meadow	++	Yes
*Lolium perenne/*‘Perennial ryegrass’	Poaceae/Pooideae	Adelphia, New Jersey, USA	Meadow	+++	Yes
*Poa annua/*‘Annual bluegrass’	Poaceae/Pooideae	Adelphia, New Jersey, USA	Meadow	+++	Yes
*Poa pratensis* ‘Kentucky bluegrass’	Poaceae/Pooideae	Adelphia, New Jersey, USA	Meadow	+++	Yes
*Phalaris arundinacea* ‘Reed canary grass’	Poaceae/Pooideae	Chatsworth, New Jersey, USA	Wetland	0	No
*Secale cereale* ‘Rye’	Poaceae/Pooideae	Chatsworth, New Jersey, USA	Cultivated	0	No
*Tritucum aestivum* ‘Wheat’	Poaceae/Pooideae	Chatsworth, New Jersey, USA	Cultivated	0	No
*Sorghum bicolor/*‘Sorghum’	Poaceae/Panicoideae	New Brunswick, New Jersey, USA	Cultivated	0	No
*Zea diploperennis/*‘Teosinte’	Poaceae/Panicoideae	Tucson, Arizona, USA	Cultivated	0	No
*Zea mays/*‘Indian corn’	Poaceae/Panicoideae	New Brunswick, New Jersey, USA	Cultivated	0^2^	No
*Zea mays/*‘Sweet corn’	Poaceae/Panicoideae	New Brunswick, New Jersey, USA	Cultivated	0	No

^1^0 = no evidence of H_2_O_2_ secreted from roots, + = low intensity or lighter zones around roots, +++ is higher intensity or darker zones around roots, and ++ are intermediate intensity zones

^2^H_2_O_2_ staining in the plant tissues but not showing substantial secretion into agarose medium

## Results

### Effects of light and bacterial presence on H_2_O_2_ secretion by tall fescue seedlings

We found the highest production of H_2_O_2_ in the non-disinfected seedlings maintained under light conditions (intensity = +++). Non-disinfected seedlings maintained in dark conditions showed a reduction in H_2_O_2_ production (intensity = ++). Disinfected seedlings maintained in light conditions showed low levels of H_2_O_2_ production (intensity = +). Disinfected seedlings maintained in dark conditions showed no or negligible H_2_O_2_ production (intensity = 0). Seeds of tall fescue obtained from commercial sources yielded the same results in this experiment.

### Effects of nutrient environment on H_2_O_2_ secretion by tall fescue seedlings

The results of these experiments are summarized in Table 1. We found that the amino acids alanine, glycine and yeast extract, reduced H_2_O_2_ production at the 0.1 % concentrations as evidenced by reductions in the percentage of seedlings showing H_2_O_2_ secretion and intensity of H_2_O_2_ secretion zones when compared to agarose only controls or the 0.01 % concentrations of each nitrogen source (Table 1). None of the inorganic nitrogenous compounds that we evaluated showed notable differences in overall seedling H_2_O_2_ secretion intensity between the 0.01 and 0.1 % concentrations, although there was a slight decrease (ranging from 13 to 23 % lower than agarose alone controls) in the percentage of seedlings showing H_2_O_2_ secretion in either 0.01 % or 0.1 % levels depending on the compound (Table 1). When compared to agarose alone controls, we found that 0.1 % cellulase enzyme stimulated a marked increase in the secretion of H_2_O_2_ with dense zones forming around seedling roots (Table 1). This stimulation of H_2_O_2_ secretion was enhanced in seedlings generated from surface disinfected seeds. Media containing agarose alone gave only diffuse H_2_O_2_ zones around roots.

### Visualization of bacterial oxidation on tall fescue seedlings

**Fig. 1:**
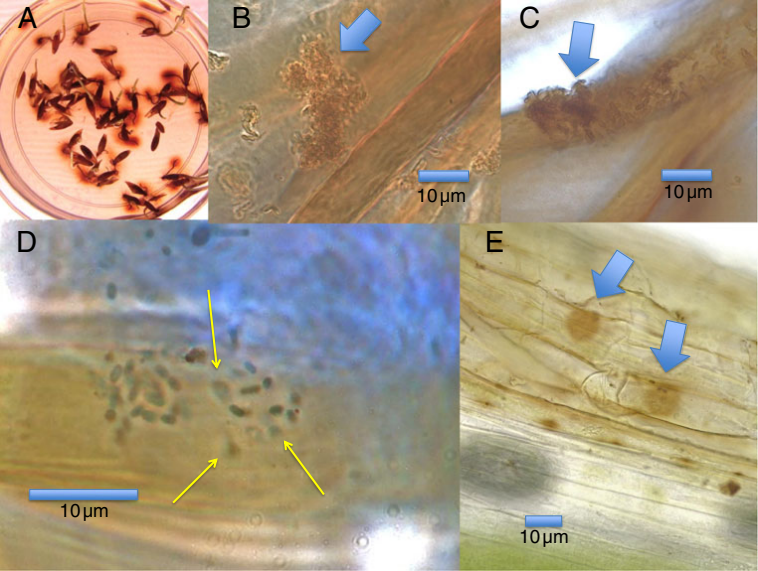
**a–e** Tall fescue seedlings stained with DAB. **a** Fescue seedlings on agarose showing H_2_O_2_ secretion (*red coloration*) from roots; **b** and **c** Fescue root hair showing bacterial cells staining brown due to H_2_O_2_ concentration (*arrows*); **d** Fescue root hair showing bacterial cells losing rod structure and capacity to stain with aniline blue during oxidation (*arrows*; stained with DAB/peroxidase, then counterstained with 0.1 % aniline blue; **e** Seedling mesocotyl showing brown areas (*arrows*) that mark sites of bacterial oxidation

**Fig. 2:**
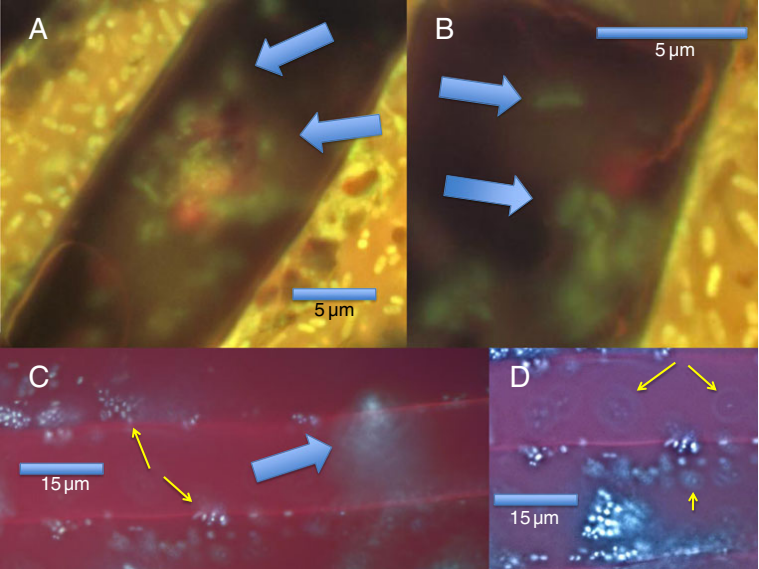
**a–d**. Root tissues and bacteria stained with SYTO9 florescent nucleic acid stain. **a** and **b** Root hairs showing epiphytic swollen oxidizing bacteria (*arrows*) with intact bacteria in background; **c** Root parenchyma showing intensely staining intact bacteria (*yellow arrows*) and masses of oxidizing bacteria (*blue arrow*); **d** Root parenchyma showing nucleic acid rings (*bulls-eye rings*) around oxidizing bacteria (*arrows*)

In microscopic examinations of seedlings using DAB/peroxidase we detected areas of high oxidation activity around bacteria. By counterstaining tissues with aniline blue, it was possible to visualize intact bacteria on plant tissues. During oxidation, bacteria lost capacity to stain with aniline blue/lactic acid. Instead, the bacteria swelled and became spherical or amorphous with diffuse walls. Eventually the bacteria vanished on the surface of plant tissues (Fig. 1). Experiments using seeds of tall fescue obtained from commercial sources gave the same results in this study. Through application of the florescent nucleic acid stain SYTO9®, we were able to observe swollen, oxidizing bacteria become amorphous, with nucleic acids dimming and diffusing away from the original site of the oxidized bacteria. We frequently visualized ‘bulls-eye rings’ around degraded bacteria (Fig. 2d). We characterize these rings as being composed of partially degraded fragments of nucleic acids that adhere to plant cell walls. More intense secretion of H_2_O_2_ in the vicinity of the bacterial cells may result in the clearing of nucleic acids nearest to the original bacterial sites, thus producing the ‘bulls-eye ring’ effect.

### Nitrogen assimilation by tall fescue seedlings

In ^15^N_2_ gas assimilation experiments (Table 2), mass spectroscopic analysis showed that tall fescue seedlings incorporated ^15^N into tissues. Greatest incorporation was observed in seedlings that had not been surface sterilized in all three runs of the experiment. Incorporation of ^15^N into seedlings seemed to correlate with the amount of 15N_2_ gas placed into the experimental chambers. Seeds or seedling material that had not been exposed to ^15^N_2_ gas showed minimal ^15^N content, ranging from 0.64 to 2.02 delta over air. Agarose media analyzed was not found to contain detectible nitrogen and ^15^N/^14^N ratios could not be calculated.

### Survey of grass species for seedling root secretion of H_2_O_2_ and bacterial oxidation

Hydrogen peroxide secretion from seedling roots was seen in pooid grasses *Bromus tectorum, Festuca arundinaceae, Festuca ovina, Lolium perenne, Poa annua* and *Poa pratensis*. Bacteria on seedling root hairs were often observed to oxidize as described in 3.3, above. Other grasses screened, including *Phalaris arundinacea, Secale cereale, Triticum aestivum, Sorghum bicolor, Zea diploperennis* and *Zea mays* did not show H_2_O_2_ secretion in agarose media. Data are summarized in Table 3.

## Discussion

### Bacterial induction of H_2_O_2_ secretion by seedlings

We repeatedly isolated *Pantoea agglomerans* from non-disinfected tall fescue seeds. However, a *Pseudomonas* sp*.* was also occasionally isolated. It is evident that seed coats of the line of tall fescue that we employed in these experiments harbor a mixed population of Proteobacteria. Because many bacteria are capable of colonization of the exterior surfaces of grass plants, many different bacteria could be present on grass seed coats and their adherent lemmas and paleas. *Pantoea agglomerans* fixes nitrogen and has previously been implicated as a plant growth promoting bacterium (Feng et al. 2006; Yashiro et al. 2011; Johnston-Monje and Raizada 2011). Several diazotrophic pseudomonads that closely match sequences of our fescue seed isolate have also been shown to be associated with the rhizospheres of grasses (Behrendt et al. 2003). Both *P. agglomerans* and *Pseudomonas* sp. were capable of growth on Norris Nitrogen-Free Agar (data not shown), suggesting that both are diazotrophic. During germination of the seed, bacteria were seen to colonize seedling roots and shoots. Our experiments show that plant seedling root tissues secrete H_2_O_2_ and perhaps other ROS that oxidize bacterial cells, their nucleic acids and likely proteins. After reduction of bacterial populations on surfaces of seeds using sodium hypochlorite disinfection, seedlings grown on agarose medium free of any nutrients appeared to show reduced secretion of H_2_O_2_. However, tall fescue seedlings grown in agarose medium amended with heat inactivated cellulase enzyme demonstrated enhanced secretion of H_2_O_2_ into the medium around seedling roots (Table 1). We conducted similar experiments using 0.1 % (w/v) Albumen Fraction V (from bovine serum; Merck Chemicals, Darmstadt, Germany) and this protein similarly enhanced H_2_O_2_ secretion from seedlings (data not shown). This suggests that enhancement of H_2_O_2_ secretion by proteins may occur regardless of the specific protein used. Bacteria on the surface of plant seedlings may secrete enzymes to degrade cellulosic cell walls, pectins, or other components of the plant (Jayani et al. 2005). These enzymes may then be deactivated and degraded by ROS secreted by the grass seedling.

### Nitrogen fixation by microbes on grass seedlings

In the three ^15^N_2_ gas assimilation experiments involving tall fescue seedlings, we found that bacteria on or within seedlings assimilated ^15^N_2_ gas (Table 2). Mass spectroscopic analysis showed that tall fescue seedlings (delta ^15^N vs air = 17.05 to 385.67) assimilated ^15^N_2_ gas when compared to seeds and seedling controls that had not been exposed to ^15^N_2_ gas (delta ^15^N vs air = 0.64–2.02). Because nitrogenase enzymes typically function under low oxygen conditions, it is possible that bacteria fix nitrogen on the seed coat during early seedling expansion before photosynthetic activities commence (Dakora et al. 2008) or during dark periods when ROS secretion may be lower. Alternatively some bacteria may adapt to presence of ROS by formation of resistant biofilms or production of ROS scavengers to shelter nitrogenase enzymes (Watson and Schubert 1969). Surface disinfection of seeds reduced incorporation of ^15^N into seedlings but it did not eliminate it (Table 2). Johnston-Monje and Raizada (2011) reported that *Pantoea agglomerans* is endophytic in numerous ancestral and modern lines of corn; and Feng et al. (2006) reported that this bacterium is endophytic in rice. Because of these reports, it seems possible that this bacterium may become endophytic in tall fescue. However, we did not visualize it within seedling tissues. It is also possible that some surface bacteria may escape the surface disinfection procedure by embedding in the seed coat or forming dry biofilms in layers under or within paleas and lemmas. Continued presence of some diazotrophic bacteria on or within tall fescue seedlings could account for the ^15^N delta over air of 17.05 to 214.09 in seedlings from surface sterilized seeds, which suggests significant nitrogen fixation over that seen in the non-15N enriched seed and seedling controls (delta over air = 0.64 to 2.02).

### Factors affecting hydrogen peroxide secretion by seedlings

Our experiments suggest that secretion of H_2_O_2_ by tall fescue seedlings may be a regulated process. In experiments summarized in Table 1, we observed that seedlings generated from surface disinfected seeds with reduced levels of external seed coat bacteria appeared to secrete less H_2_O_2_ from roots than seedlings from seeds that were not surface disinfected. This outcome suggests that plants may be responding to the presence of bacteria by secreting ROS proportional to the amount of bacteria on plants. This conclusion is further supported by ROS activity visualization on seedling shoot tissues where we observed staining for ROS activity only at sites of bacterial oxidation (Fig. 1e).

Factors such as light and nutrient environment of seedlings may also impact H_2_O_2_ secretion by seedlings. We observed that seedlings grown under dark conditions produced less H_2_O_2_ than seedlings maintained under light conditions. Reduction of ROS secretion under dark conditions might permit bacteria to more efficiently employ oxygen-sensitive nitrogenase enzymes to fix atmospheric nitrogen at night while high ROS secretion in light may result in greater harvesting of the fixed nitrogen. In another experiment (Table 1), we observed that the amount of ROS secreted by seedlings could be reduced or stopped completely by inclusion 0.1 % of organic forms of nitrogen (e.g., amino acids or yeast extract) into the media around seedlings, suggesting that H_2_O_2_ secretion may show feedback control where seedling tissues with sufficient organic nitrogen discontinue ROS secretion. Inorganic forms of nitrogen, even at very low levels (0.01 %), tended to reduce the number of seedlings showing H_2_O_2_ secretion (Table 1). This may be an indication that inorganic nitrogen application to plants may negatively impact the proposed oxidative system for acquisition of organic nitrogen from microbes. In experiments where we disinfected seeds to decrease bacterial populations, yeast extract and amino acids reduced H_2_O_2_ secretion from roots. The full significance of these observations will require additional experimentation using inorganic and organic sources of nitrogen. However, taken collectively, the results summarized in Table 1 support the hypothesis that the function of H_2_O_2_ secretion by seedlings relates to organic nitrogen acquisition.

### ROS effects on bacterial cells

Visualization of bacterial oxidation on plant tissues suggests that oxidation does not immediately involve complete oxidation to inorganic forms. Instead, a partial oxidation of bacteria and their constituent compounds occurs, likely denaturing cell membranes and walls. Disarticulation of larger organic compounds, like proteins and nucleic acids, into smaller fragments follows. This phenomenon may be evidenced by bulls-eye rings around oxidizing bacteria when the nucleic acid probe SYTO9® (Fig. 2) is applied. The bulls-eye rings may result from diffusion of nucleic acid fragments and their adsorption to plant cell walls. More intense secretion of H_2_O_2_ in the vicinity of the bacterial cell may result in clearing of nucleic acids nearest the original site of the bacterium resulting in the bulls-eye-ring effect.

Investigations into the effects of ROS on bacterial cells are consistent with our observations and hypotheses (Cabiscol et al. 2000). In a review on ROS damage to bacterial cells, Cabiscol et al. (2000) report that nucleic acids, proteins and lipids are impacted by ROS. H_2_O_2_ produces highly reactive hydroxyl radicals via the Fenton reaction that occurs in the presence of metal ions. Lipids in bacterial walls and membranes are major targets where polyunsaturated fatty acids are peroxidized and degraded. The decomposition of the cell walls of bacteria, composed of lipids and proteins, result in loss of the cell shape. In nucleic acids ROS attacks both the nitrogenous base and sugar moieties, producing single- and double-strand breaks in the nucleic acid backbone. Proteins are denatured with alterations to amino acids and frequently show peptide fragmentation (Cabiscol et al. 2000). ROS denatured proteins show enhanced susceptibility to protease degradation (Galek et al. 1990). Van der Valk and Van Loon (1988) demonstrated that cell walls of oat leaves possess extracellular acidic proteases and Godlewski and Adamczyk (2007) demonstrated that plants secrete proteases from their roots. Similar extracellular proteases in tall fescue seedlings could contribute to the process of protein degradation on seedling roots and shoots. This process could occur regardless of whether bacteria are epiphytic on plant tissues or are endophytes within tissues.

### Role of ROS in oxidation/digestion of microbes and their proteins for defense and nutrition

Generally, scientists consider plant-secreted ROS, such as H_2_O_2,_ to be destructive metabolic byproducts of cellular respiration and photosynthesis that plants must manage internally to prevent cellular damage. Certain ROS moieties also function as signaling molecules, mediating defensive or other plant reactions (D’Autréaux and Toledano 2007). Foreman et al. (2003) demonstrated that H_2_O_2_ is produced by plasmalema NADPH oxidases and may be secreted into apoplastic spaces *in planta* (see also Dunand et al. 2007). Frahry and Schopfer (1998) demonstrated that H_2_O_2_ is secreted from roots of soybean (*Glycine max*). The proposal that ROS plays a role in oxidation of plant microbes or their protein products to extract nitrogen is a new concept in plant biology that will undoubtedly require additional confirmation. However, in animals, ROS are known to be involved in the killing and degradation action of animal phagocytic leukocytes in oxygen-dependent defense (Robinson 2008). In phagocytic leukocytes the killing and degradation action of H_2_O_2_ results from its capacity to form more potent ROS moieties, including hydroxyl radicals, singlet oxygen, ozone (Robinson 2008). A similar process could also be occurring on or within plant tissues. It is known that plants also secrete H_2_O_2_ defensively in ‘oxidative bursts’ at sites of pathogen colonization (Lamb and Dixon 1997). This defensive ROS is known to have a direct deleterious effect in killing microbial pathogens. ROS has additionally been shown to be involved in nutritive phagocytosis in starfish and other simple animals (Coteur et al. 2002). In carnivorous pitcher plants, plant-secreted ROS plays a nutritional role in digestion of insect proteins (Chia et al. 2004). Galek et al. (1990) similarly reported that the ‘venus flytrap’ (*Dionaea muscipula*) employs ROS to pre-digest insect proteins prior to protease action on the denatured peptide fragments.

We hypothesize that the oxidation process occurring on seedlings is comparable to that seen in some forms of phagocytosis in animals and nutritive digestion as seen in carnivorous plants. We suggest that ROS secreted from seedling tissues function as ‘digestive agents’ that play a role in degradation of microbes and their protein products to obtain nitrogen for the plant host. Proteases secreted by plant roots (Godlewski and Adamczyk 2007) or maintained in plant cell walls (Van der Valk and Van Loon 1988) may contribute to the process of microbial protein degradation. Amino acid or oligopeptide transporters in plant roots may transport products into root cells (Kielland 1994; Jamtgard et al. 2008). Even without the action of protease enzymes, ROS may be effective in oxidizing proteins to smaller oligopeptides or amino acids that may be assimilated by the seedlings (Hill et al. 2011). Investigations by Kocha et al. (1997) demonstrated that hydrogen peroxide-mediated protein degradation occurs without the presence of proteases or other catalyzing enzymes. Through H_2_O_2_-mediated non-specific degradation of proteins, smaller peptide fragments and eventually nitrates (Palenik and Morel 1990) may form on seedling tissue surfaces. Bacterial proteins may provide a significant amount of the nitrogen needed by developing grass seedlings. When it is considered that rapidly growing seedlings have a high requirement for nitrogen and that their roots are not yet fully developed for efficient nitrogen absorption from soils, it is not unexpected that some plants would evolve a mechanism to scavenge nitrogen from the diazotrophic bacteria that rapidly colonize seedlings during germination. To describe the process whereby plants may oxidize microbes or their proteins to obtain nitrogen we propose the term ‘oxidative nitrogen scavenging’ (ONS).

### Grass species that show ONS by seedlings

In our survey (Table 3) of grass seedlings to identify additional species that show ONS, the phenomenon was most notable in several grass species of subfamily Pooideae, including *Bromus tectorum, Festuca arundinaceae, Festuca ovina, Lolium perenne, Poa pratensis* and *Poa annua*. *Phallaris arundinacea*, also subfamily Pooideae, did not show H_2_O_2_ secretion. The highly selected and long cultivated grasses wheat, rye, sorghum and corn did not show evidence of H_2_O_2_ secretion (Table 3). In species where we report the phenomenon of seedling H_2_O_2_ secretion and bacterial oxidation (Table 3), seeds possess adherent plant tissues (dry paleas and lemmas) that disperse with the seeds. It is possible that seeds are colonized by bacteria on their paleas and lemmas during the period of maturation prior to their release from plants. Thus, seed coat adherent paleas and lemmas may function to vector bacteria to seedlings. *Phallaris arundinacea*, wheat, rye, sorghum and corn possess seeds that are free of adherent paleas and lemmas and thus may not be adapted to vector bacteria of their seed coats. It is possible that ancestral forms of wheat, rye, sorghum and corn possessed seed-transmitted diazotrophic microbes but that these have been lost in cultivation. However, if ancestors of crop species possessed the capacity for ONS it may be possible to improve modern crops by re-establishing these symbioses to reduce the need for external nitrogen inputs.

Many of the grasses that we have identified as showing enhanced H_2_O_2_ secretion from roots are known for their competitive abilities and have come to be used as forage, conservation or turf grasses. These species include the fescues (*Festuca arundinaceae* and *F. ovina*), perennial ryegrass (*Lolium perenne*), annual bluegrass (*Poa annua*) and Kentucky bluegrass (*Poa pratensis*). *Poa annua* and *Bromus tectorum* are both generally aggressive and often ‘invasive’ (Sperry et al. 2006; Chwedorzewska 2008). It is possible that the competitive abilities of these grass species may be, at least in part, due to their capacity to extract nutrients from bacteria. Further, oxidative systems to extract nutrients from microbes may not be limited to grasses. Many other plants are known to thrive under conditions where nitrogen is difficult or impossible to extract from soils. Future work will need to evaluate the existence and importance of oxidative nitrogen scavenging systems in enabling plants in diverse families to grow in nitrogen limited habitats.

## Conclusions

The proposal that some plants possess a system to extract nitrogen from microbes through oxidation is new, but not unreasonable given that plants possess the basic metabolic machinery (e.g., NADPH oxidases for ROS generation, proteases, oligopeptide and amino acid transporters) needed for such a process (Kielland 1994; Godlewski and Adamczyk 2007; Jamtgard et al. 2008). Further, many plants are known to be rich sources of diazotrophic microbes that inhabit much of their exterior and interior surfaces (Arnold and Lutzoni 2007; Reinhold-Hurek and Hurek 2011). A capacity of some plants to directly extract nitrogen from symbiotic bacteria would alter our longstanding view of the role of these ubiquitous microbes in and on those plants. Rather than being casual or opportunistic inhabitants of plants, diazotrophic microbes may represent a reservoir of fixed nitrogen that plants cultivate when available soil nitrogen is abundant and harvest through oxidation in circumstances when nitrogen cannot be extracted from the soil. Definitive validation of ONS in plants would also alter our view of how plants gain nutrients in demonstrating ‘microbivory’ as an option for nutrient acquisition. This is a nutritional mode where typically small animals or protists consume bacteria as a nutrient source (Mikola 1998). We are not the first investigators to suggest that plants may show microbivory. Paungfoo-Lonhienne et al. (2010) presented evidence that some plants appear to phagocytize bacteria as a nutrient source. Confirmation of the functioning and importance of the proposed ONS mechanism will require additional experimentation to track the flow of nitrogen into cells and tissues and evaluate the precise roles of ROS, plant proteases and plant cell transporters in the degradation and absorption process.
